# Stearic Acid and TNF-α Co-Operatively Potentiate MIP-1α Production in Monocytic Cells via MyD88 Independent TLR4/TBK/IRF3 Signaling Pathway

**DOI:** 10.3390/biomedicines8100403

**Published:** 2020-10-09

**Authors:** Shihab Kochumon, Hossein Arefanian, Rafaat Azim, Steve Shenouda, Texy Jacob, Nermeen Abu Khalaf, Fatema Al-Rashed, Amal Hasan, Sardar Sindhu, Fahd Al-Mulla, Rasheed Ahmad

**Affiliations:** 1 Immunology & Microbiology Department, Dasman Diabetes Institute, Kuwait City 15462, Kuwait; shihab.kochumon@dasmaninstitute.org (S.K.); hossein.arefanian@dasmaninstitute.org (H.A.); rafaatazim27@gmail.com (R.A.); steve.shenouda@dasmaninstitute.org (S.S.); texy.jacob@dasmaninstitute.org (T.J.); fatema.alrashed@dasmaninstitute.org (F.A.-R.); amal.hasan@dasmaninstitute.org (A.H.); 2 School of Medicine, Royal College of Surgeons in Ireland, Medical University of Bahrain, Adliya 15503, Bahrain; 3 Animal & Imaging Core Facility, Dasman Diabetes Institute, Kuwait City 15462, Kuwait; nermeen.abukhalaf@dasmaninstitute.org (N.A.K.); sardar.sindhu@dasmaninstitute.org (S.S.); 4 Genetics and Bioinformatics, Dasman Diabetes Institute, Kuwait City 15462, Kuwait; fahd.almulla@dasmaninstitute.org

**Keywords:** MIP-1α, stearic acid, TNF-α/CCL3, TLR4, TRIF/TBK, IRF3, obesity

## Abstract

Increased circulatory and adipose tissue expression of macrophage inflammatory protein (MIP)-1α (CC motif chemokine ligand-3/CCL3) and its association with inflammation in the state of obesity is well documented. Since obesity is associated with increases in both stearic acid and tumor necrosis factor α (TNF-α) in circulation, we investigated whether stearic acid and TNF-α together could regulate MIP-1α/CCL3 expression in human monocytic cells, and if so, which signaling pathways were involved in MIP-1α/CCL3 modulation. Monocytic cells were treated with stearic acid and TNF-α resulted in enhanced production of MIP-1α/CCL3 compared to stearic acid or TNF-α alone. To explore the underlying mechanisms, cooperative effect of stearic acid for MIP-α/CCL3 expression was reduced by TLR4 blocking, and unexpectedly we found that the synergistic production of MIP-α/CCL3 in MyD88 knockout (KO) cells was not suppressed. In contrast, this MIP-α/CCL3 expression was attenuated by inhibiting TBK1/IRF3 activity. Cells deficient in IRF3 did not show cooperative effect of stearate/TNF-α on MIP-1α/CCL3 production. Furthermore, activation of IRF3 by polyinosinic-polycytidylic acid (poly I:C) produced a cooperative effect with TNF-α for MIP-1α/CCL3 production that was comparable to stearic acid. Individuals with obesity show high IRF3 expression in monocytes as compared to lean individuals. Furthermore, elevated levels of MIP-1α/CCL3 positively correlate with TNF-α and CD163 in fat tissues from individuals with obesity. Taken together, this study provides a novel model for the pathologic role of stearic acid to produce MIP-1α/CCL3 in the presence of TNF-α associated with obesity settings.

## 1. Introduction

The incidence of obesity is growing worldwide. The presence of obesity leads to the development of chronic inflammation, which is critical in the pathogenesis of insulin resistance and metabolic syndrome [1]. Chronic overnutrition results in obesity, which causes systemic metabolic and inflammatory abnormalities in the adipose tissue. Adipose tissue is a central metabolic organ in the regulation of whole-body energy homeostasis and plays a key role in the obesity-induced inflammation called metabolic inflammation [2]. Metabolic inflammation involves increased expression of adipokines such as leptin and visfatin as well as proinflammatory cytokines such as interleukin 1-β (IL-1β), tumor necrosis factor α (TNF-α), interleukin 6 (IL-6), interleukin 8 (IL-8), and interleukin 12 (IL-12), most of which are implicated in insulin resistance and type 2 diabetes (T2D) [3,4]. TNF-α is also shown to be involved in glucose homeostasis and lipolysis in adipocytes [5,6,7]. Besides cytokines/chemokines produced by immune cells in adipose tissue, adipocyte-driven metabolites such as saturated free fatty acids (FFAs) are also potent mediators of chronic inflammation and insulin resistance in obesity [8]. Individuals with obesity have elevated levels of different plasma FFAs, which mainly consist of palmitic acid and stearic acid [8]. Along with palmitic acid, stearic acid is the primary substrate for the enzyme stearoyl-coenzyme A (stearoyl-CoA) desaturase, which converts stearic acid into oleic acid used for the synthesis of triglycerides (TG) and other complex lipids [9]. The role of palmitic acid and stearic acid in lipid metabolism is well defined. Palmitic acid is a stimulator of toll-like receptor 4 (TLR4) signaling pathways in monocytes/macrophages and contributes to obesity-related chronic inflammation and insulin resistance [10]. However, stearic acid role in the regulation of inflammatory responses in monocytic cells is poorly studied.

In both humans and rodents, the accumulation of monocytes/macrophages into the adipose tissue correlates with the development of obesity and insulin resistance [3]. Chemokines regulate the trafficking and infiltration of circulatory monocytes and other immune effector cells into adipose tissue. Macrophage inflammatory protein (MIP) MIP-1α/CCL3 (CC motif chemokine ligand-3/CCL3) is a chemoattractant for monocytes, neutrophils, basophils, NK cells and T cells, and MIP-1α/CCL3 is produced by monocytes, macrophages, T lymphocytes, NK cells, dendritic cells, and other cell types [11,12]. Although elevation in circulatory and adipose expression of MIP-1α/CCL3 in metabolic syndrome is documented [13,14,15,16,17], the production and regulation of MIP-1α/CCL3 remains to be elucidated. As higher levels of stearic acid and TNF-α have been observed in the circulation of obese/type 2 diabetic individuals, therefore, we asked whether stearic acid and TNF-α could cooperatively enhance the MIP-1α/CCL3 production in monocytic cells and adipocytes. In this report, we demonstrate that incubation of human monocytic cells with stearic acid results in enhanced production of MIP-1α/CCL3 upon exposure to TNF-α via Myeloid differentiation factor 88 (MyD88) independent TLR4/ Interferon regulatory factor 3 (IRF3) signaling pathway.

## 2. Experimental Section

### 2.1. Reagents, Antibodies, and Cell Lines

Recombinant human TNF-α (cat#: 210-TA-100) were obtained from R&D systems (Minneapolis, MN, USA). Lipopolysaccharides (LPS) (cat#: L4391), Phorbol 12-myristate 13-acetate (PMA) (cat#: P1585), Stearic acid (Cat#: W303518), Poly I:C (cat#: P9582), chlorpromazine (cat#: C8138), and resveratrol (cat#: R5010) were purchased from Sigma (San Diego, CA, USA). Quanti-Luc medium (cat#: rep-qlc1), Quanti-blue medium (cat#: rep-qb-2), and BX795 (cat#: tlrl-bx7), were purchased from InvivoGen (San Diego, CA, USA). SP600125 (cat#: 8177), U0126 (cat#: 9903), cell lysis buffer (cat#: 9803) were obtained from Cell Signaling Technology (Danvers, MA, USA). TLR4 (ID 7099), Trilencer-27 Human siRNA (SR322051), scrambled (control) siRNA (SR30004), and IRF3 (cat#: ID 3661) Trilencer-27 Human siRNA (SR320690) were obtained from OriGene (Rockville, MD, USA). The Antibiotics Blasticidin (cat#: ant-bl), HygroGold (cat#: ant-hg), Zeocin (cat#: ant-zn) and Normocin (cat#: ant-nr) were purchased from InvivoGen (San Diego, CA, USA). GA-1000 SingleQuots™ was purchased from Lonza (Walkersville, MD, USA). TLR4 neutralizing antibody (cat#: mabg-htlr4) and Immunoglobulin A2 (IgA2) isotype control (cat#: maba2-ctrl) were purchased from InvivoGen (San Diego, CA, USA). IRF-3 (cat#: D83 B9), Rabbit mAb (cat#: 4302), Phospho-IRF-3 (Ser396) (cat#: 4947) were obtained from Cell Signaling Technology (Danvers, MA, USA). Human Subcutaneous Primary preadipocyte cells (cat#: PT-5001) were purchased from Lonza (Walkersville, MD, USA). Human monocytic leukemia THP-1 cell line was purchased from American Type Culture Collection (ATCC, Manassas, VA, USA). THP1-Dual™ knockout (KO)-MyD Cells (cat#: thpd-komyd) and THP-1-XBlue™-defMyD cells were purchased from InvivoGen (San Diego, CA, USA).

### 2.2. Preparation of Stearic Acid

Briefly, a 120 mM solution of stearic acid in ethanol was prepared and complexed with 1 mM fatty acid-free Bovine Serum Albumin (BSA) prepared in serum-free culture media at 1:20 *v*/*v* ratio to make a final concentration of 6.1 mM stearic acid-BSA conjugate. The complex was mixed and sonicated at 37 °C for 45 min in a water bath with sonicator. The solution was filter sterilize using a 0.22 µM syringe filter. A similar concentration of ethanol-BSA conjugate (without stearic acid) was also prepared to be used as vehicle for control treatments.

### 2.3. Cell Culturing and Stimulation

THP-1 and primary monocytes (1 × 10^6^ cells/mL) were cultured using 12-well plates (Costar, Corning Incorporated, Corning, NY, USA) in Roswell Park Memorial Institute 1640 (RPMI-1640) medium (Gibco, Life Technologies, Grand Island, NY, USA) containing 10% fetal bovine serum (FBS) (Gibco, Life Technologies, Grand Island, NY, USA), 2 mM glutamine, 1 mM sodium pyruvate, 10 mM HEPES, 100 µg/mL Normocin, 50 U/mL penicillin and 50 μg/mL streptomycin and cells were incubated at 37 °C in 5% CO_2_ under humidity. THP-1-XBlue™-defMyD cells were cultured in complete RPMI medium containing Zeocin (200 µg/mL) and HygroGold (100 µg/mL). THP1-Dual™ KO-MyD cells were grown in complete RPMI medium containing Normocin™ (100 μg/mL), Blasticidin (10 μg/mL) and Zeocin™ (100 μg/mL) to select for cells expressing the secreted embryonic alkaline phosphatase (SEAP)- Nuclear factor-κB (NF-κB)/ Activator protein 1 (AP-1) or Luciferase Interferon regulatory factor (IRF)/ serum responsive element (SRE) reporter.

### 2.4. Human Subcutaneous Primary Adipocyte Culture and Differentiation

Human Subcutaneous Primary adipocytes (0.1 × 10^6^ cells/mL) were cultured using 12-well plates (Costar, Corning Incorporated, Corning, NY, USA) in complete Preadipocyte Growth Medium-2 (PGM-2, cat#: PT-8202; Lonza, Alpharetta, GA, USA) supplemented with FBS (10%), L-glutamine (2 mM), GA-1000 SingleQuots™ (Gentamicin; 30 µg/mL and Amphotericin; 15 ng/mL), and cells were then incubated for 24 h at 37 °C in 5% CO_2_. Preadipocyte differentiation into adipocytes was induced with the addition of equal volume of 2× Adipocyte Differentiation Medium (complete PGM-2 medium containing insulin, indomethacin, isobutyl-methylxanthine, and dexamethasone) to each well and incubated for 9 days at 37 °C in 5% CO_2_ until the cells became fully differentiated by the formation of intracellular lipid vacuoles. Differentiated adipocytes were treated with stearic acid and/or TNF-α for 24 h. Cells were harvested for RNA isolation and supernatants were collected for MIP-1α/CCL3 quantification.

### 2.5. Isolation of Peripheral Blood Mononuclear Cells (PBMC), Primary Monocyte Purification and Stimulation

Human peripheral blood (40 mL) were collected in ethylenediaminetetraacetic acid (EDTA) vacutainer tubes from healthy donors at the Dasman Diabetes Institute (DDI) and following written informed consent of participants and study approval by the research ethics committee of DDI. PBMC were isolated using HistoPaque density gradient method as described elsewhere [18]. Monocytes were isolated from the PBMCs using indirect magnetic colloid labeling method (Monocyte Isolation Kit II; cat#:130-091-153 Miltenyi Biotec, Germany) in which non-monocytic cells including T cells, NK cells, B cells, dendritic cells, and basophils were magnetically labeled with cocktail of biotin-conjugated antibodies against CD3, CD7, CD16, CD19, CD56, CD123, and CD235a (Glycophorin A) surface antigens and anti-biotin MicroBeads. Purified CD14^+^ monocytes were eluted through the magnetic column and purity was determined by flow cytometry [19]. Cells were stimulated with stearic acid (200 μM; Sigma) and/or TNF-α (10 ng/mL; Sigma) or 0.1% BSA and incubated at 37 °C for 24 h. Later, cells were harvested for total RNA isolation and culture media were collected for secreted MIP-1α/CCL3 measurement.

### 2.6. Collection of Subcutaneous Adipose Tissue Samples

A total of 10 lean and 18 individuals with obesity were recruited in the study at the Dasman Diabetes Institute clinics. All participants were given a written informed consent. Studies were approved by the ethics committee of Dasman Diabetes Institute, Kuwait and conducted in compliance with the Declaration of Helsinki. The clinicodemographic data of the study participants are summarized in Supplementary Table S1. Subcutaneous adipose tissue samples (~0.5 g) were collected through fat pad biopsy lateral to the umbilicus using standard surgical technique. Briefly, periumbilical area was sterilized and locally anesthetized using 2% lidocaine (2 mL). After making a small superficial skin incision (0.5 cm), the subcutaneous fat tissue was collected and further incised into small pieces. Samples were rinsed in cold phosphate buffered saline (PBS), and freshly collected adipose tissue samples (~50–100 mg) were preserved in RNAlater and stored at −80 °C until use [20].

### 2.7. Cell Stimulation and Chemokine/Cytokine Measurements

Primary human monocytes, THP-1 cells, IRF reporter cells (THP1-Dual™ KO-MyD Cell) and THP-1-XBlue™-defMyD cells were cultured (1 × 10^6^ cells/well) in 12-well plates and stimulated with stearic acid (200 μM), TNF-α (10 ng/mL), and stearic acid/TNF-α combined, for 24 h. Cells were harvested for RNA isolation, and supernatants were collected and stored at −80 °C for quantification of MIP-1α/CCL3 or Luc/SEAP activity. MIP-1α/CCL3 was quantified in cell supernatants using Human DuoSet enzyme-linked immunosorbent assay (ELISA) Kit (R&D systems, Minneapolis, MN, USA) as per manufacturer’s instructions.

### 2.8. Real-Time Quantitative Reverse Transcription Polymerase Chain Reaction (RT-PCR)

Total cellular RNA was extracted using RNeasy Mini Kit (Qiagen, Valencia, CA, USA) and cDNA was synthesized from 1 μg of total RNA using high capacity cDNA reverse transcription kit (Applied Biosystems, Foster City, CA, USA). For real-time PCR, cDNA (50 ng) was amplified using TaqMan Gene Expression Assay reagents (GAPDH: Hs03929097_g1; MIP-1α: Hs00234142_m1; TNF-α: Hs01113624_g1; IRF3: Hs01547283_m1; TLR4: Hs00152939_m1; CD163: Hs00174705_m1) containing two gene-specific primers, one TaqMan MGB probe (6-FAM dye-labeled) and TaqMan^®^ Gene Expression Master Mix, using a 7500 Real-Time PCR System (Applied Biosystems, Foster City, CA, USA). The mRNA levels were normalized against GAPDH mRNA and the expression of MIP-1α/CCL3 mRNA relative to control was calculated using the 2^-ΔΔCt^ method [21]. Relative mRNA expression was shown as fold expression over average of control gene expression taken as 1 and data were presented as mean ± SEM values.

### 2.9. Gene Silencing

Gene silencing was performed using the transient transfection method, Amaxa Cell Line Nucleofector Kit V (Lonza, Germany), and Amaxa Electroporation System (Amaxa Inc., Cologne, Germany), as per manufacturer’s instructions. For transient transfection, THP-1 cells (1 × 10^6^ cells/mL) were resuspended in Nucleofector^®^ solution and transfected separately using 30 nM IRF3 siRNA, TLR4 siRNA and scrambled negative control siRNA. After 36 h, transfected cells were treated with stearic acid (200 μM), TNF-α (10 ng/mL), or stearic acid/TNF-α combined, for 24 h. Cells were harvested for RNA isolation and supernatants were collected for MIP-1α/CCL3 quantification. In addition, real time-PCR was performed to confirm the effective suppression of constitutive IRF3 or TLR4 expression in THP-1 cells transfected with IRF3 siRNA or TLR4 siRNA and scrambled negative control siRNAs.

### 2.10. Luciferase Assay

THP1-Dual™ KO-MyD cells were cultured in normal growth medium without any selective antibiotics and treated with stearic acid, TNF-α, or stearic acid/TNF-α combined for 24 h. IRF3 activity, induced by Stearate or TNF-α treatment, was then assessed by measuring Luciferase activity in the supernatants. This was achieved by mixing 50 μL of QUANTI-Luc luciferase assay reagent (InvivoGen, San Diego, CA, USA) with 10 μL of sample in a 96-well black plate. The light signal produced proportional to IRF3 activity was then quantified using a luminometer set at 0.1 s reading time and expressed as relative light units (RLU).

### 2.11. Western Blotting

Human PBMCs or THP-1 cells were incubated for 30 min with lysis buffer containing Tris (62.5 mM; pH 7.5), 1% Triton X-100 (Sigma, San Diego, CA, USA), and 10% glycerol (Sigma, San Diego, CA, USA). Cell lysates were centrifuged at 14,000 rpm for 10 min, supernatants were collected, and protein was measured using Quickstart Bradford Dye Reagent and 1 × Protein Assay kit (Bio-Rad Laboratories, Hercules, CA, USA). Samples (20 μg) were mixed with loading buffer, heated at 95 °C for 5 min and resolved by 12% SDS-PAGE. Resolved proteins were transferred to immunoblot polyvinylidene difluoride (PVDF) membrane (Bio-Rad Laboratories, USA) by electroblotting, blocked with 5% non-fat milk in PBS for 1 h, and then incubated overnight at 4 °C with primary antibodies (1:100 dilution; Cell Signaling Technology Inc. Danvers, MA, USA) against IRF3 and P-IRF3. Blots were washed 4 times with Tris Buffered Saline (TBS) buffer and incubated for 2 h with Horseradish Peroxidase (HRP)-conjugated secondary antibody (Promega, Madison, WI, USA). Immunoreactive bands were developed using an Amersham ECLPlus Western Blotting Detection System (GE Health Care, Buckinghamshire, UK) and visualized using Molecular Imager^®^ VersaDoc™ MP Imaging System (Bio-Rad Laboratories, Hercules, CA, USA).

### 2.12. Statistical Analysis

The data obtained were expressed as mean  ±  SEM values and group means were compared using unpaired *t*-test. The linear dependence between two variables was assessed by Pearson’s correlation coefficient (r). GraphPad Prism software (Version 6.05; San Diego, CA, USA) was used for statistical analysis as well as for graphical representation of the data. All *p*-values ≤ 0.05 were considered as statistically significant.

## 3. Results

### 3.1. Stearic Acid Cooperatively Increases TNF-α Mediated MIP-1α/CCL3 Production

**Figure 1:**
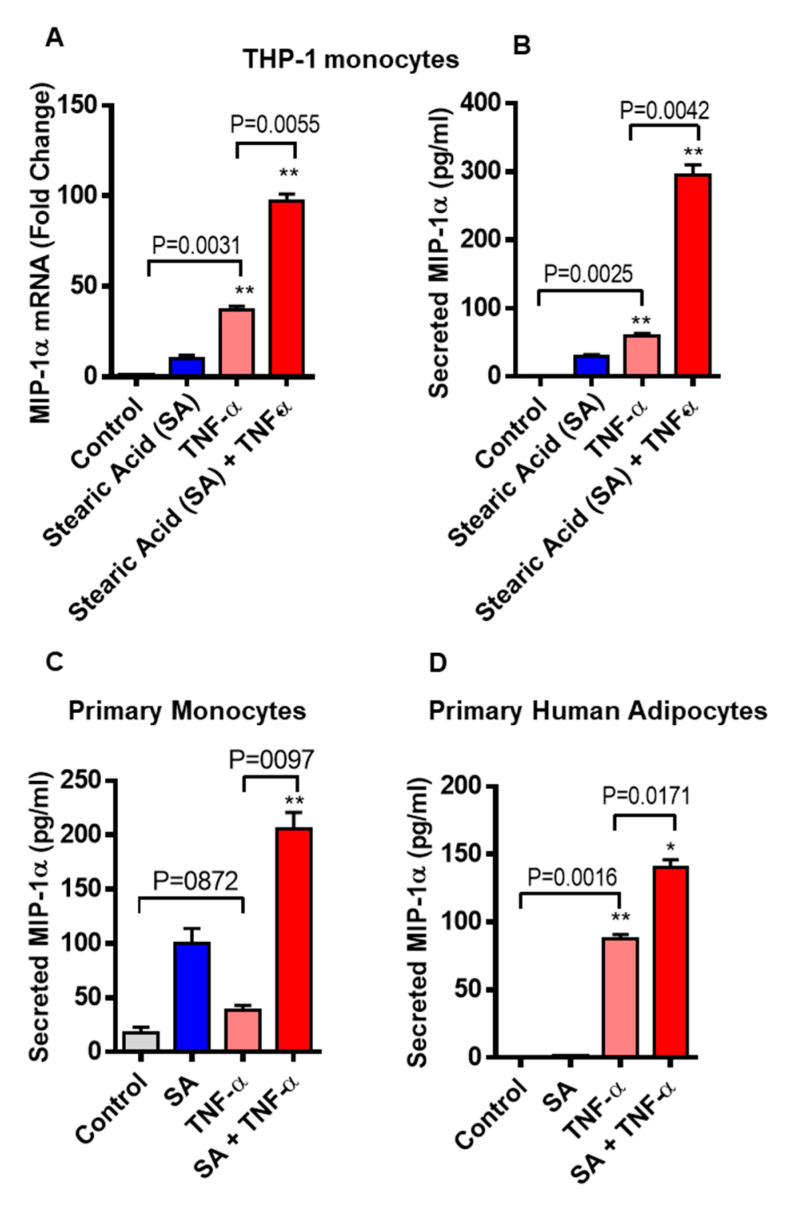
Co-treatment of stearic acid and tumor necrosis factor α (TNF-α) enhances macrophage inflammatory protein (MIP)-1α (CC motif chemokine ligand-3/CCL3) production. (**A**) THP-1 cells were incubated with stearic acid (200 µM) and TNFα (10 ng/mL) for 24 h. Cells and culture media were collected. Total RNA was extracted from the cells and *CCL3* mRNA was quantified by real time PCR. Relative mRNA expression was expressed as fold change (**B**) Secreted MIP-1α/CCL3 protein in culture media was determined by ELISA. (**C**,**D**) Primary human monocytes and adipocytes were treated with stearic acid and TNF-α for 24 h. *MIP-1α/CCL3* mRNA and secreted protein were determined. The results obtained from minimum three independent experiments with three replicates of each experiment are shown. All data are expressed as mean ± SEM (*n* ≥ 3); * *P* < 0.05; ** *P* < 0.01, when compared with stearic acid or TNF-α alone.

We asked whether stearic acid has a cooperative effect on TNF-α mediated MIP-1α/CCL3 production in monocytic cells. We found that cotreatment of TNF-α and stearic acid synergize the MIP-1α/CCL3 gene expression in monocytic cells (THP-1 cells and primary human monocytes) compared to cells treated with either TNF-α or stearic acid alone. (Figure 1A). Similarly, MIP-1α/CCL3 protein expression was also higher in stearic acid/TNF-α treated cells (Figure 1B). The cooperative effect of stearic acid with TNF-α on THP-1 monocytic cells was a concentration-dependent manner with a maximal production of MIP-1α/CCL3 at its concentration of 200 uM (Supplementary Figure S1). A similar relationship was observed between stearic acid and TNF-α in primary human monocytes (Figure 1C). Given these observations in the monocytes, we next wanted to know if similar cooperative effects of stearic acid/TNF-α were also occurring in human primary adipocytes. To this end, experiments on human adipocytes showed stearic acid cooperatively triggered TNF-α mediated MIP-1α/CCL3 production (Figure 1D). It was also noted that stimulation of THP-1 cells, primary monocytes and primary human adipocytes with TNF-α alone induced significantly higher gene/protein expression of MIP-1α/CCL3 (Figure 1A–D). Altogether, these results demonstrate the stearic acid cooperative effect plays a major role in triggering the TNF-α mediated production of MIP-1α/CCL3 by human monocytes and adipocytes.

### 3.2. Inhibition of TLR4 Signaling Disrupts the Cooperative Effect of Stearic Acid/TNF-α on MIP-1α/CCL3 Production

**Figure 2:**
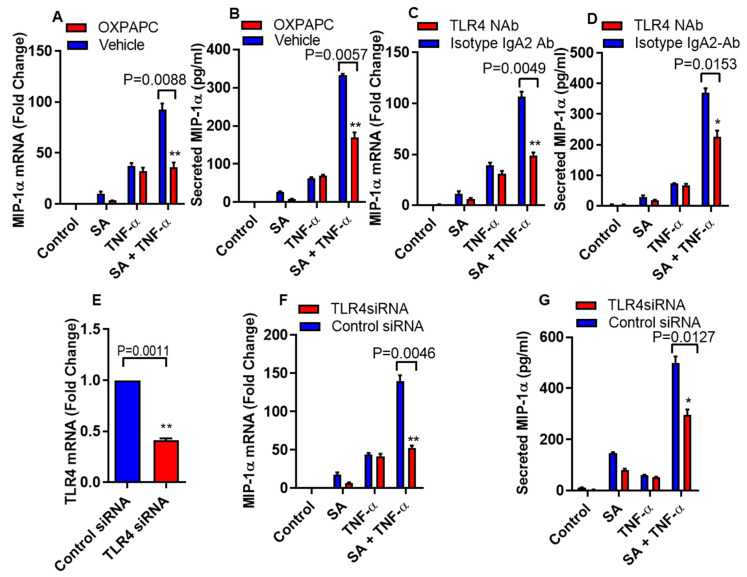
Disruption of TLR4 suppresses cooperative effect of stearic acid with TNF-α for MIP-1α/CCL3 production. (**A**,**B**) THP-1 cells were incubated with oxidized 1-palmitoyl-2-arachidonyl-sn- glycero-3-phosphorylcholine (OXPAPC) (30 µg/mL) for 1 h and then treated with stearic acid and TNF-α for 24 h. Cells and culture media were collected. MIP-1α/CCL3 gene expression was determined by real time PCR and secreted MIP-1α/CCL3 protein was determined in culture media by ELISA. (**C**,**D**). Monocytic cells were treated with 2 µg/mL of neutralizing TLR4 mAb or isotype-matched control (IgA2) for 40 min. Antibody-treated cells were treated with stearic acid and TNF-α for 24 h. MIP-1α/CCL3 mRNA and secreted MIP-1α/CCL3 protein were determined. (**E**–**G**) Monocytic cells were transfected with either control or TLR4 siRNA. TLR4-deficient cells were stimulated with stearic acid and TNF-α for 24 h. Knockdown efficiency of transfection was checked by TLR4 gene expression with qRT-PCR. MIP-1α/CCL3 mRNA and secreted MIP-1α/CCL3 protein were determined. The results obtained from minimum three independent experiments with three replicates of each experiment are shown. All data are expressed as mean ± SEM (*n* ≥ 3); * *P* < 0.05; ** *P* < 0.01when compared with Stearic acid or TNF-α alone.

Inflammatory mediators are regulated by FFAs through the activation of TLR4 signaling pathways [22]. To define the role of TLR4 signaling in the cooperative effect of stearic acid/TNF-α in relation to the production of MIP-1α/CCL3, THP-1 monocytic cells were treated with oxidized 1-palmitoyl-2-arachidonyl-sn- glycero-3-phosphorylcholine (OXPAPC), a chemical inhibitor specific for TLR4 or vehicle, before the treatment with stearic acid, TNF-α alone or in combination. Inhibition of TLR4 with OXPAPC suppresses the stearic acid/TNF-α cooperative effect on MIP-α/CCL3 gene expression at both mRNA and protein levels (Figure 2A,B). However, as expected, OXPAPC did not show any effect on TNF-α mediated production of MIP-1α/CCL3 (Figure 2A,B). To further confirm the role of TLR4 in stearic acid cooperative effect on MIP-1α/CCL3 production, THP-1 monocytic cells were pretreated with TLR4 neutralizing antibody or a control isotype. Neutralization of TLR4 on monocytic cells led to suppress cooperative effect of stearic acid for TNF-α mediated MIP-1α/CCL3 production compared to the cells pretreated with the control isotype antibody (Figure 2C,D). Furthermore, small interfering RNA mediated knockdown leads to the same conclusion, demonstrating that cooperative effect of stearic acid on the TNF-α mediated MIP-α production requires TLR4 signaling (Figure 2E–G).

### 3.3. Cooperative Effect of Stearic Acid on TNF-α Mediated MIP-1α/CCL3 Production Is MyD88-Independent and IRF3-Dependent Pathways

**Figure 3:**
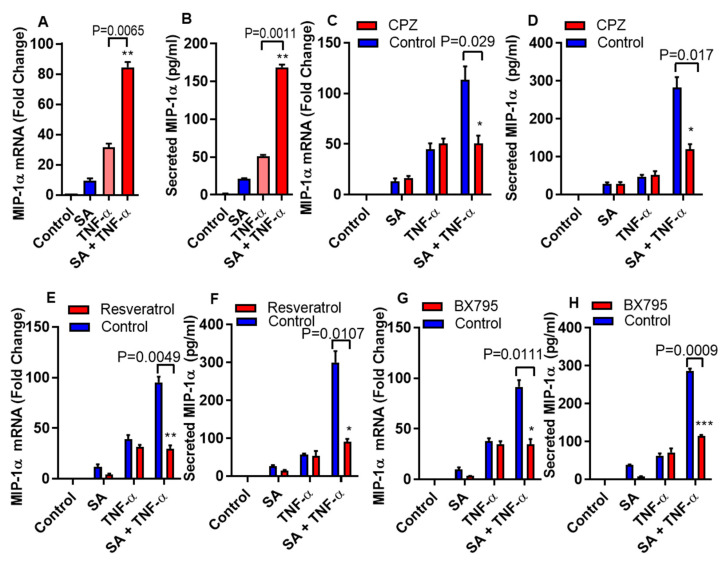
Stearic acid cooperative effect on MIP-1α/CCL3 is MyD88 independent and dependent on TRIF/TBK1. (**A**,**B**) Cells deficient in MyD88 activity (THP-1 defMyD cells) were treated with stearic acid (200 µM), 0.1% BSA (control) or TNF-α (10 ng/mL) alone or in combination. Cells and culture media were collected after 24 h. CCL3 mRNA and secreted MIP-1α/CCL3 protein were determined. (**C**,**D**) Monocytic cells were pretreated with chlorpromazine (CPZ; an inhibitor of endocytosis; 10 µM) for 1 h and then treated as indicated for 24 h. MIP-1α/CCL3 mRNA and secreted MIP-1α/CCL3 protein were determined. (**E**,**F**) Cells were treated with resveratrol (a TRIF inhibitor; 5 uM) for 30 min followed by treatments as indicated. MIP-α expression was determined. (**G**,**H**) Cells were incubated with BX795 (an inhibitor for TBK1/IKKε; 100 nM) for 1 h and then treated for 24 h as indicated. MIP-1α/CCL3 expression was determined. The results obtained from minimum three independent experiments with three replicates of each experiment are shown. All data are expressed as mean ± SEM (n ≥ 3); * *P* < 0.05; ** *P* < 0.01; *** *P* < 0.001, when compared with stearic acid or TNF-α alone.

MyD88 or TRIF/IRF3 dependent signal pathways can activate under the influence of TLR4 mediated inflammatory responses [23,24,25]. To determine whether the cooperative effect of stearic acid on TNF-α mediated production of MIP-α requires MyD88, we used MyD88 null (-/-) THP-1 cells. We found that MyD88 deficiency did not block the priming effect of stearic acid on the production of MIP-1α/CCL3 (Figure 3A,B). Adaptor proteins TIR domain–containing adapter-inducing IFN-β (TRIF)/ TANK-binding kinase 1 (TBK1) associates with TLR4 after clathrin-dependent internalization and leads to the activation of IRF3 and its downstream signaling cascade [26,27]. Next, we found that the cooperative effect of stearic acid was inhibited by blocking clathrin-dependent endocytosis (Figure 3C,D).

Furthermore, we confirmed the involvement of TRIF in this priming effect of stearic acid using small molecule (Resveratrol) inhibitor. Pretreatment of monocytic cells with resveratrol abrogated the cooperative actions of stearic acid on the TNF-α mediated production of MIP-1α/CCL3 (Figure 3E,F). Similarly, inhibiting the TBK1 adaptor protein with BX795 attenuates the cooperative effect of stearic acid on MIP-1α/CCL3 production (Figure 3G,H).

**Figure 4:**
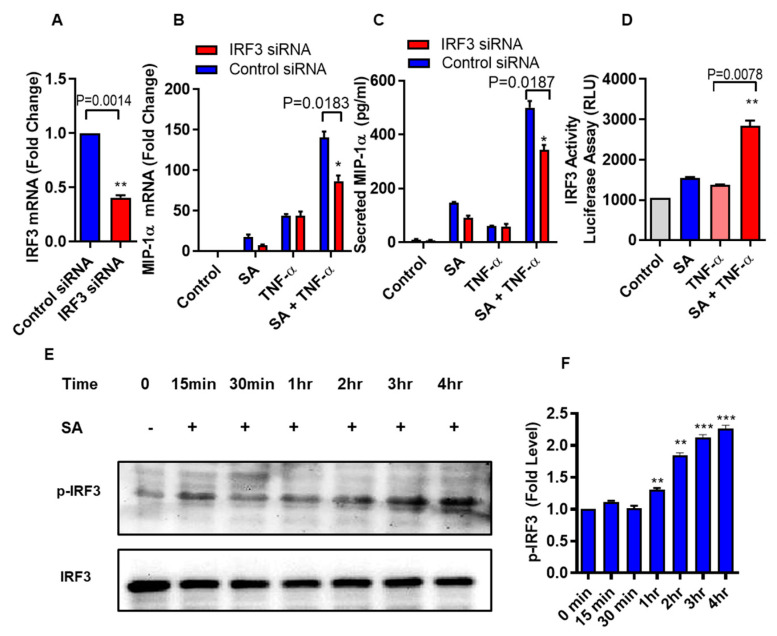
Stearic acid cooperative effect with TNF-α for MIP-1α/CCL3 production requires IRF3. (**A**) THP-1 monocytic cells were transfected with either control or IRF3 siRNA and incubated for 36 h. Real time PCR was done to measure IRF3 expression. (**B**,**C**) IRF3 deficient THP-1 cells were stimulated with stearic acid and TNF-α. *MIP-1α/CCL3* expression was determined. The results obtained from minimum three independent experiments with three replicates of each experiment are shown. (**D**) IRF3 activity reporter monocytic cells were treated with stearic acid (200 µM) or 0.1% BSA (control) or TNF-α (10 ng/mL) or in combination. Culture media were collected after 24 h. Cell culture media were assayed for luciferase activity representing the degree of IRF3/ISRE activation using Quanti-Luc medium. (**E**) Western blot analysis showed that stearic acid induced IRF3 phosphorylation in a time dependent manner in THP-1 monocytes, verifies the role of IRF3 in the cooperative effect of stearic acid in the TNF-α mediated production of MIP-1α/CCL3. (**F**) Expression of phosphorylated IRF3 is shown as determined by densitometry of western blot bands. * *P* < 0.05; ** *P* < 0.01; *** *P* < 0.001.

Next, we verify that the cooperative effect of stearic acid on TNF-α mediated production of MIP-1α/CCL3 was dependent on the TLR4-IRF3 signaling axis. THP-1 Monocytic cells were transfected with *Irf3* siRNA and scrambled siRNA control (Figure 4A). Cooperative effect of stearic acid/TNF-α on MIP-1α/CCL3 production was significantly reduced in the IRF3 deficient THP-1 cells (Figure 4B,C). IRF3 reporter cells showed that stearic acid enhances the IRF3 activity (Figure 4D). Western blot analysis showed that stearic acid induced IRF3 phosphorylation in a time-dependent manner, verifies the role of IRF3 in the cooperative effect of stearic acid for TNF-α mediated production of MIP-1α/CCL3 (Figure 4E).

**Figure 5:**
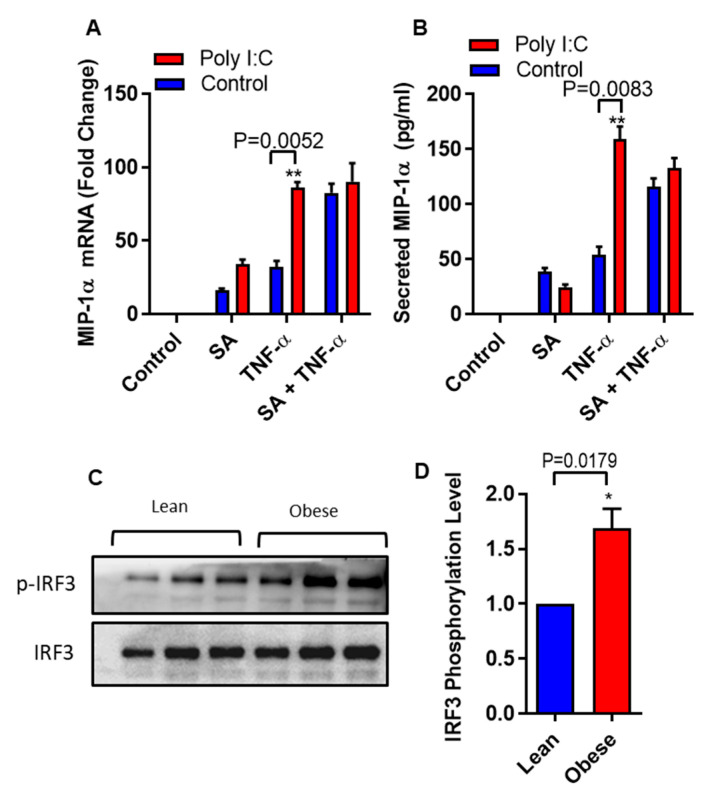
Polyinosinic-polycytidylic acid (poly I:C) act as a substitute of stearic acid cooperative effect on MIP-1α/CCL3 production. (**A**,**B**) THP-1 cells were treated (via transfection) with poly I:C (5 µg) for 2 h and then incubated with BSA (control) or stearic acid or TNF-α for 24 h. MIP-1α/CCL3 mRNA and protein were determined. The results obtained from minimum three independent experiments with three replicates of each experiment are shown. All data are expressed as mean ± SEM (*n* ≥ 3); * *P* < 0.05; ** *P* < 0.01, when compared with stearic acid or TNF-α alone. (**C**,**D**) Western blot analysis showed that individuals with obesity have significant higher levels of phospho-IRF3 in monocytes compared to lean individuals (*P* = 0.0179).

It is well documented that IRF3 can be activated by polyinosinic-polycytidylic acid (poly I:C) [28]. We asked whether we could circumvent the need of stearic acid by specific activation of IRF3 using polyinosinic-polycytidylic acid (poly I:C) in THP-1 monocytic cells. Poly I:C- mediated IRF3 activation generated a similar cooperative effect on TNF-α induced MIP-1α/CCL3 production as in case of stearic acid (Figure 5A,B). Next, we also found that individuals with obesity have high levels of phospho-IRF3 in THP-1 monocytes compared to lean (Figure 5C,D). Overall, these genetic and pharmacologic lines of evidence strongly support the assumption that TLR4/MyD88 independent signaling pathway is the major, if not the only, mediator of the cooperative effect of stearic acid on the TNF-α–induced activation of MIP-1α/CCL3 gene expression.

### 3.4. Stearic Acid and TNF-α Cooperatively Activates NF-kB

**Figure 6:**
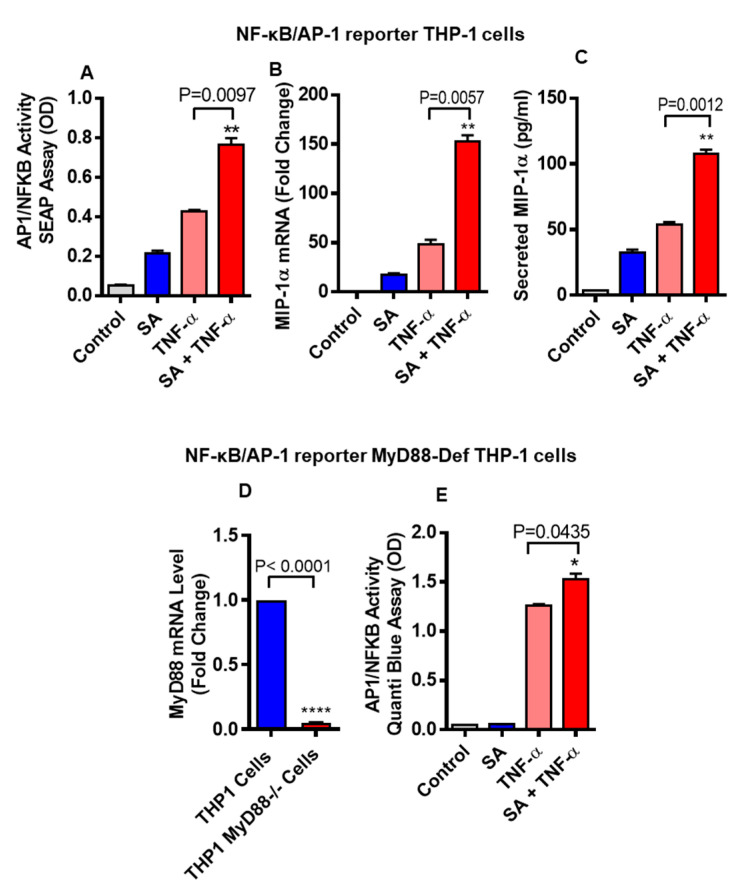
THP-1-XBlue cells (THP-1 cells stably expressing a secreted embryonic alkaline phosphatase (SEAP) reporter inducible by NF-κB and AP-1) were treated with stearic acid or BSA or TNF-α for 24 h. Culture media were collected. Cell culture media were assayed for SEAP reporter activity (degree of NF-κB/AP-1 activation) (**A**), along with MIP-1α gene and protein expression (**B**,**C**). Next, THP-1-XBlue™-defMyD cells (Cells deficient in MyD88 activity) were used showing defective gene expression of MyD88 (**D**). These cells were also treated with stearic acid (200 μM), TNF-α (10 ng/mL) and 0.1% BSA for 24 h and SEAP reporter activity (degree of NF-κB/AP-1 activation) is shown (**E**). The results obtained from three independent experiments are shown. The data are presented as mean ± SD. * *P* < 0.05; ** *P* < 0.01; **** *P* < 0.001, when compared with stearic acid or TNF-α alone.

It has been well documented that NF-κB/AP-1 signaling pathway is activated downstream to TLR4 and TNF-α receptor (TNFR) when these receptors get activated. To test whether stearic acid and TNF-α cooperatively activates NF-κB/AP-1 signaling, NF-κB/AP-1 reporter monocytic cells were used. Our results show that these cells co-stimulated with stearic acid and TNF-α show significantly higher expression of MIP-1α/CCL3 compared to cells treated with stearic acid or TNF-α alone (Figure 6A). At the same time, we measured gene and protein expression of MIP-1α/CCL3 in these cells, showing that MIP-1α/CCL3 expression was significantly higher in cells co-treated with stearic acid and TNF-α compared to controls treated with stearic acid or TNF-α alone (Figure 6B,C). Furthermore, we used MyD88-defective cells (MyD88 deficient expression shown in Figure 6D) and found that stearic acid and TNF-α synergy did not depend on presence of MyD88 adaptor protein (Figure 6E).

### 3.5. MIP-1α/CCL3 Is Associated with TNF-α in Subcutaneous Fat from Humans with Obesity

**Figure 7:**
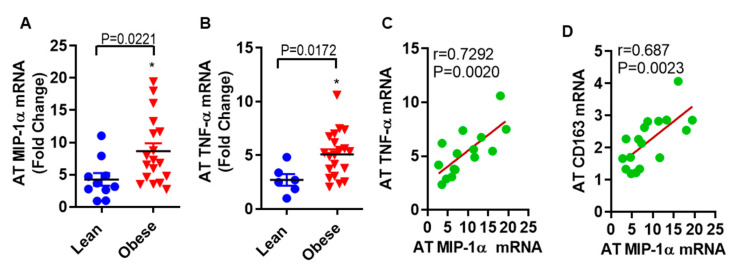
MIP-1α/CCL3 is associated with TNF-α in subcutaneous fat from obese humans. The level of (**A**) MIP-1α/CCL3 (8.67 ± 1.22 folds, *p* = 0.0221) and (**B**) TNF-α (5.09 ± 0.48 folds, *p* = 0.0172) mRNA levels were significantly higher in obese as compared to lean individuals (4.28 ± 0.99 folds for MIP-1 α, and 2.72 ± 0.54 folds for TNF-α). In obese adipose tissue, a strong positive correlation was found between (**C**) MIP-1α/CCL3 and TNF-α mRNA expression (r = 0.7292; *p* = 0.0022), and (**D**) MIP-1α/CCL3 and CD163 (r = 0.686, *p* = 0.0023).

The findings from in vitro work with human primary subcutaneous adipocytes led us to ask whether the observed association exists between MIP-1α/CCL3 and TNF-α in subcutaneous fat samples from obese as compared with lean individuals. In fat tissue, MIP-1α/CCL3 (8.67 ± 1.22 folds, *p* = 0.0221) and TNF-α (5.09 ± 0.48 folds, *p* = 0.0172) mRNA levels were significantly higher in obese as compared to lean individuals (4.28 ± 0.99 folds for MIP-1 α, and 2.72 ± 0.54 folds for TNF-α, Figure 7A,B). Interestingly, in obese adipose tissue, a strong positive correlation was found between MIP-1α/CCL3 and TNF-α mRNA expression (r = 0.7292; *p* = 0.0022, Figure 7C). MIP-1α/CCL3 correlates positively with CD163 (r = 0.686, *p* = 0.0023, Figure 7D).

**Figure 8:**
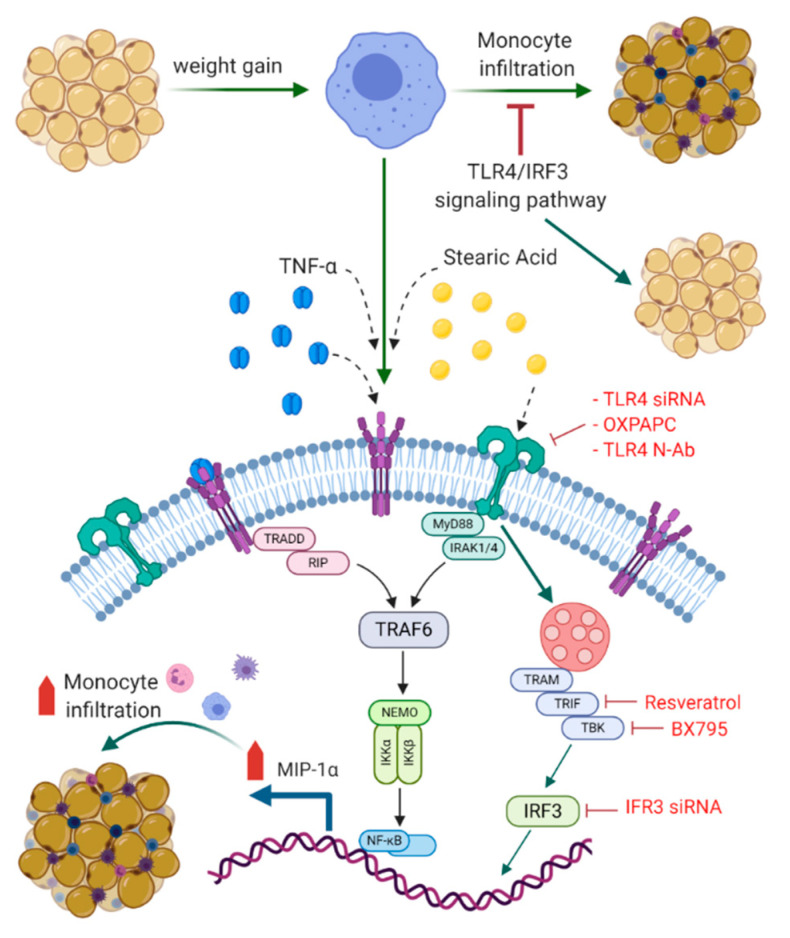
Schematic illustration of signaling pathways underlying the cooperative relationship between stearic acid and TNF-α for MIP-1α/CCL3 production. Blocking TLR4/IRF3 signaling pathways significantly suppress the cooperative production of MIP-1α/CCL3 by stearic acid/TNF-α. TLR: Toll like receptor; TNF-α: Tumor necrosis factor α; MyD88: Myeloid differentiation factor 88; IRAK: Interleukin-1 receptor-associated kinase; TRIF: TIR domain–containing adapter-inducing IFN-β; IRF3: Interferon regulatory factor-3; TBK: TANK-binding kinase 1; TRAM: Toll-receptor-associated molecule; TRAF6: tumor-necrosis factor receptor-associated factor 6: IKK-Iκ-B kinase; NF-kB: Nuclear factor-κB; TRADD: Tumor necrosis factor receptor 1-associated death domain protein; RIP: receptor-interacting protein; NEMO: NF-kappa-B essential modulator. BioRender.com was used for Figure 8.

To summarize the underlying signaling pathways involved in this cooperative relationship between stearic acid and TNF-α for MIP-1α/CCL3 production, a schematic illustration is presented in Figure 8.

## 4. Discussion

This study demonstrates, for the first time, that the cooperative effect of stearic acid and TNF-α mediates the production of MIP-1α/CCL3 in human monocytic cells and adipocytes. Stearic acid levels along with TNF-α are increased in the plasma of individuals with obesity [8,29,30]. Thus, it is likely that stearic acid plays a role in the TNF-α mediated induction of inflammatory marker MIP-1α/CCL3 in the settings of obesity. Saturated free fatty acids (FFAs) are well-documented stimulators of monocytes/macrophages and contribute to obesity-related chronic inflammation and insulin resistance [22,31,32,33,34,35]. Stearic acid potentiated the production of VEGF, IL-6, and other proinflammatory cytokines expression in primary mouse chondrocytes [36]. Furthermore, we identified the role of TLR4 signaling pathways in the cooperative effect of stearic acid on the TNF-α mediated production of MIP-1α. The previous studies support that saturated free fatty acids exert proinflammatory roles through the TLR4 in multiple cell types including monocytes, macrophages, adipocytes and chondrocytes [22,36,37,38,39,40,41]. TLR4-downstream signaling involves either MyD88-or TRIF/TBK1 related adaptor molecules [42,43]. Verstak et al. reported a novel role for Toll-receptor-associated molecule (TRAM) in TLR4-mediated signaling via its interaction with tumor-necrosis factor receptor-associated factor 6 (TRAF6), distinct from its role as a bridging adaptor between TLR4 and TRIF [44]. It is noteworthy to test the involvement of these adaptor molecules. Our results showed that MyD88 deficiency did not block the cooperative effects of stearic acid on MIP-1α/CCL3 production. Though MyD88 is a critical adaptor protein for TLR4 signaling related to inflammation [42,45,46]. TLR4 uses TRIF/TBK1/IKKε as other adaptor molecules to induce the expression of IFN-β in a MyD88-independent manner [43]. Interestingly, our data showed that disturbing the activity of these molecules inhibits the cooperative effect of stearic acid to produce MIP-1α/CCL3. These results suggest that MyD88 independent pathway is indispensable for the cooperative effect of stearic acid.

IRF3 is one of the major effectors of TLR4-MyD88 independent signaling pathway. Activation of IRF3 upregulates the expression of interferons and several inflammatory genes [47,48,49]. Our result showed that IRF3 deficiency significantly blocks the cooperative effect of stearic acid. Furthermore, IRF3 activation by poly-IC mimics the cooperative effect of stearic acid on the production of MIP-1α/CCL3 by TNF-α. Individuals with obesity have elevated levels of stearic acid along with other fatty acids. We found that individuals with obesity have a high expression of p-IRF3 compared to lean individuals, suggesting that plasma free fatty acids in individuals with obesity contribute to the activation of proinflammatory genes regulated under the influence of IRF3. Overexpression of IRF3 in the adipocytes in both human and mouse obesity has been reported. It was shown that activation of TLR4/IRF3 signaling pathways results in insulin resistance in murine adipocytes. Furthermore, mice lacking IRF3 are protected from diet-induced insulin resistance and systemic inflammation. Thus, IRF3 deficiency enhances the browning of subcutaneous fat along with increased adipose expression of GLUT4 [28]. These data confirm the role of IRF3 as a primary transcriptional regulator involve in adipose tissue inflammation, maintaining systemic glucose, and energy homeostasis. Our data show that stearic acid/TNF-α synergy involves the NF-κB/AP-1 mediated signaling. However, it is also plausible that stearic acid accumulation in macrophages may trigger inflammation independent of TLR4 involvement via the ER/oxidative stress [50].

Our human adipose tissue data show high expression of MIP-1α/CCL3 and TNF-α in obese compared to lean individuals, and these markers are positively correlated in obesity state. Obese adipose tissue of humans displays increased expression of MIP-1α, TNF-α, and macrophage markers [13,14,15,16]. Adipose tissue macrophages are the most abundant immune cell population in fat and linked to obesity-induced inflammation in both mouse models and humans [51,52]. Our data shows that MIP-1α/CCL3 correlates with macrophage marker along with TNF-α, which reflects the infiltration of macrophages into inflamed adipose tissues. Thus, we speculate that simultaneously increased plasma levels of free fatty acid, TNF-α and MIP-1α/CCL3 may have the potential to induce significant pathophysiological changes in the adipose tissue compartment in obesity, including changes in insulin sensitivity and lipid metabolism.

## 5. Conclusions

In summary, our results show that cooperative effect of stearic acid on the TNF-α induced production of MIP-1α/CCL3 depends on TLR4-TRIF-TBK1-IRF3 signaling cascade which suggests an interesting pathophysiological link among stearic acid, TNF-α, and MIP-1α/CCL3 in the state of obesity.

## Supplementary Materials

The following are available online at https://www.mdpi.com/2227-9059/8/10/403/s1, Figure S1: Dose dependent manner of cooperative effect of stearic acid on TNF-α mediated MIP-1α/CCL3 production. Table S1: Characteristics of the study participants title.
