# Supplementary material for: Stearic Acid and TNF-α Co-Operatively Potentiate MIP-1α Production in Monocytic Cells via MyD88 Independent TLR4/TBK/IRF3 Signaling Pathway
Source: Biomedicines. 2020 Oct 9;8(10):403. doi: 10.3390/biomedicines8100403 (PMC7600458; doi:10.3390/biomedicines8100403)
Supplement: Supplementary file 1 [file biomedicines-08-00403-s001.pdf]

Fig:S1

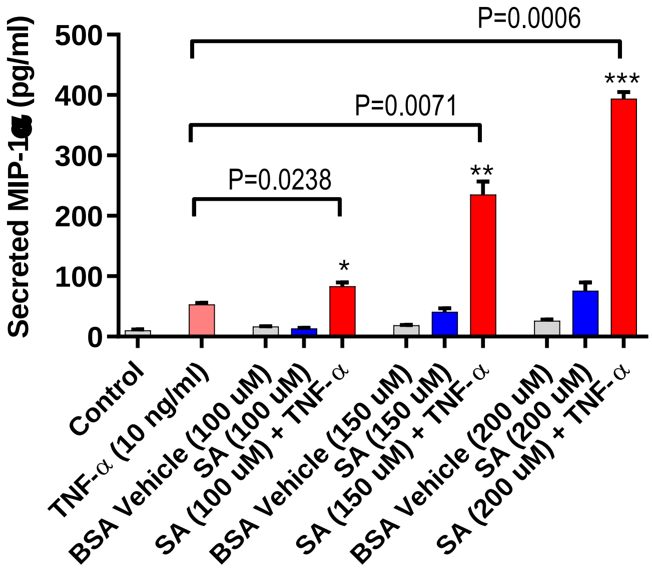

Supplemental Figure 1: Dose dependent manner of cooperative effect of stearic acid on TNF-α mediated MIP-1α production. THP-1 cells were treated with different concentrations of stearic acid (100 µM, 150 µM, 200 µM) and then treated with TNFα (10 ng/ml) for 24 h. Culture media were collected. Secreted MIP-1α protein in culture media was determined by ELISA.

Supplemental Table 1: Characteristics of the study participants

Phenotype	Lean	Obese
	(n = 10) (Mean ± SD)	(n = 18) (Mean ± SD)
Age (years)	42.7 ±8.17	48.89± 14.19
Weight (kg)	62.93 ± 11.90	93.39 ± 14.14
Height (cm)	1.66 ± 0.12	1.65 ± 0.09
BMI (kg/m2)	22.82 ± 2.35	34.69 ± 3.64
Waist (cm)	81.33 ± 12.44	106.74 ± 12.86
Body fat (%)	28.37 ± 6.27	39.53 ± 3.95
FBS (mmol/L)	4.97 ± 0.64	5.43± 0.81
TGL (mmol/L)	0.63 ± 0.24	1.30 ± 0.92
Chol (mmol/L)	5.3 ± 1.11	5.14± 1.21
HDL (mmol/L)	1.69 ± 0.51	1.27 ± 0.31
LDL (mmol/L)	3.31 ± 0.93	3.31 ± 0.98
HbA1c (%)	5.66 ± 0.46	5.65 ±0.74
Insulin (mU/L)	6.58 ± 3.34	16.50 ±12.42
HOMA-IR	1.40± 0.64	5.12 ±4.46
WBC	5.57± 1.60	5.87 ±1.66
